# Ophiopogonin D promotes bone regeneration by stimulating CD31^hi^EMCN^hi^ vessel formation

**DOI:** 10.1111/cpr.12784

**Published:** 2020-02-20

**Authors:** Mi Yang, Chang‐Jun Li, Ye Xiao, Qi Guo, Yan Huang, Tian Su, Xiang‐Hang Luo, Tie‐Jian Jiang

**Affiliations:** ^1^ Department of Endocrinology, Endocrinology Research Center Xiangya Hospital of Central South University Changsha China

**Keywords:** bone regeneration, CD31^hi^EMCN^hi^ vessel, Krüppel like factor 3, Ophiopogonin D

## Abstract

**Objectives:**

CD31^hi^EMCN^hi^ vessels (CD31, also known as PECAM1 [platelet and endothelial cell adhesion molecule 1]; EMCN, endomucin), which are strongly positive for CD31 and endomucin, couple angiogenesis and osteogenesis. However, the role of CD31^hi^EMCN^hi^ vessels in bone regeneration remains unknown. In the present study, we investigated the role of CD31^hi^EMCN^hi^ vessels in the process of bone regeneration.

**Materials and Methods:**

We used endothelial‐specific Krüppel like factor 3 (*Klf3*) knockout mice and ophiopogonin D treatment to interfere with CD31^hi^EMCN^hi^ vessel formation. We constructed a bone regeneration model by surgical ablation of the trabecular bone. Immunofluorescence and micro‐computed tomography (CT) were used to detect CD31^hi^EMCN^hi^ vessels and bone formation.

**Results:**

CD31^hi^EMCN^hi^ vessels participate in the process of bone regeneration, such that endothelial‐specific *Klf3* knockout mice showed increased CD31^hi^EMCN^hi^ vessels and osteoprogenitors in the bone regeneration area, and further accelerated bone formation. We also demonstrated that the natural compound, ophiopogonin D, acts as a KLF3 inhibitor to promote vessels formation both in vitro and in vivo. Administration of ophiopogonin D increased the abundance of CD31^hi^Emcn^hi^ vessels and accelerated bone healing.

**Conclusions:**

Our findings confirmed the important role of CD31^hi^Emcn^hi^ vessels in bone regeneration and provided a new target to treat bone fracture or promote bone regeneration.

## 1 INTRODUCTION

As one of the biggest and most complex organ systems in mammals, the skeleton is full of cracks and undergoes continuous shaping, remodelling and repair throughout adulthood.^1^, ^2^, ^3^, ^4^, ^5^ Bone can self‐heal robustly in most cases; however, accelerating bone regeneration to meet clinical need would be advantageous.^1^, ^6^, ^7^, ^8^, ^9^ Bone regeneration is a highly complex and temporally coordinated process comprising various cellular and molecular processes.^2^, ^10^, ^11^, ^12^

Blood vessels transport oxygen, nutrients, and waste, and also act as a passageway for cell‐signalling molecules.^13^, ^14^, ^15^ Specialized vessels formed in different tissues participate in the formation of a specific microenvironment that decides the fate of progenitor cells.^15^, ^16^, ^17^, ^18^, ^19^, ^20^ Therefore, it is important to investigate the effects of vessels on tissue regeneration. CD31^hi^EMCN^hi^ vessels (CD31, also known as PECAM1 [platelet and endothelial cell adhesion molecule 1]; EMCN, endomucin), which are strongly positive for CD31 and endomucin, are specific vessels in the skeletal system that couple angiogenesis and osteogenesis.^18^ Our previous studies demonstrated that inducing CD31^hi^EMCN^hi^ vessels could stimulate bone formation.^21^, ^22^, ^23^ Angiogenesis coupled with osteogenesis plays an important role in bone metabolism; however, the role of CD31^hi^EMCN^hi^ vessels in bone regeneration remains unknown.

Our previous study demonstrated that mutant REG1CP (regenerating family member 1 gamma) increased the formation of the CD31^hi^Emcn^hi^ endothelium in bone marrow by binding to Krüppel‐like factor 3 (KLF3) to inhibit its activity. We also identified a natural compound, ophiopogonin D, which functions as a KLF3 inhibitor.^23^ Administration of ophiopogonin D increased the abundance of CD31^hi^Emcn^hi^ vessels and bone formation.^23^ In the present study, we expanded our research and demonstrated that ophiopogonin D acts as a KLF3 inhibitor to promote vessel formation and further stimulate bone regeneration in young (3 months old) and middle‐aged (12 months old) mice.

## 2 MATERIALS AND METHODS

### 2.1 Mice

To specific knockout *Klf3* in endothelium, we crossed mice carrying loxP‐flanked *Klf3* alleles (*Klf3^flox/flox^*) with *Cdh5‐Cre* transgenics to get *Cdh5‐Cre: Klf3^flox/flox^*mice (*Klf3*_cdh5_^−/−^). The *Klf3^flox/flox^* littermates were used as controls. The *Cdh5‐Cre* transgenic mice (Stock No. 017968) were purchased from Jackson Laboratory, and *loxP‐flanked Klf3* mice were purchased from Cyagen Biosciences Inc (China). The bone regeneration model was established as described before.^24^, ^25^ A longitudinal incision was made on each knee to expose the femoral condyle by patella dislocation. Then, we used a dental drill to make a hole was at the intercondylar notch of the femur. A 0.6‐mm‐diameter Kirschner wire was placed from the proximal end of the femur. We confirmed the bone ablation of trabecular bone by radiography. Bone samples were collected 1 week after the surgery. For ophiopogonin D treatment experiment, mice under surgical ablation of trabecular bone were intraperitoneally treated with ophiopogonin D at dosage of 20 mg/kg every day for 7 days. All mice we used were C57BL/6J background. Male mice at indicated age were used in our experiments. All mice were maintained in standard, specific pathogen‐free facility of the Laboratory Animal Research Center of Central South University.

### 2.2 Isolate primary BMSCs

We isolated primary BMSCs as reported previously.^26^, ^27^ We collected all bone marrow cells and incubated them with FITC‐, APC‐ and PE‐conjugated antibodies which recognized mouse Sca‐1 (BioLegend, 108108), CD29 (BioLegend, 102206), CD45 (BioLegend, 103132) and CD11b (BioLegend, 101226) for 30 minutes at 4°C. Then, we performed fluorescence‐activated cell sorting (FACS) and analysis the results using FACS DIVE software version 6.1.3 (BD Biosciences). The sorted mouse Sca‐1^+^CD29^+^CD45^−^CD11b^−^ BMSCs were cultured with α‐MEM (Gibco‐BRL Co.) supplemented with 10% FBS, 100 U/mL penicillin and 100 μg/mL streptomycin.

### 2.3 Osteogenic differentiation assay

Isolated BMSCs were cultured with α‐MEM (Gibco‐BRL Co.) supplemented with 10% FBS, 100 U/mL penicillin, 100 μg/mL streptomycin, 0.1 mmol/L dexamethasone, 10 mmol/L b‐glycerol phosphate and 50 mmol/L ascorbate‐2‐phosphate for 21 days. Culture medium was changed every three days. Cells were collected for RNA extraction or stained with 2% Alizarin Red S (Sigma‐Aldrich) at pH 4.2 to evaluate the cell matrix mineralization.

### 2.4 Osteoclasts differentiation assay

Osteoclasts differentiation assay was performed as reported previously.^28^ Monocytes and macrophages were collected from bone marrow of mice by flushing the marrow space of femora and tibiae. The isolated bone marrow cells were cultured with α‐MEM (Gibco‐BRL Co.) supplemented with 10% FBS, 100 U/mL penicillin, 100 μg/mL streptomycin for 12 hours; then, the floating cells were cultured with α‐MEM (Gibco‐BRL Co.) supplemented with 10% FBS, 100 U/mL penicillin, 100 μg/mL streptomycin and 30 ng/mL M‐CSF (R&D Systems Inc) for 3 days to obtain pure monocytes and macrophages. Then, these cells were cultured with α‐MEM (Gibco‐BRL Co.) supplemented with 10% FBS, 100 U/mL penicillin, 100 μg/mL streptomycin, 30 ng/mL M‐CSF and 60 ng/mL RANKL (PeproTech) for 8 days. Cells were collected for RNA extraction or stained with TRAP (Sigma‐Aldrich).

### 2.5 Migration assay

Human microvascular endothelial cells (HMECs) were cultured with MCDB131 medium (Gibco) supplemented 10% FBS, 100 U/mL penicillin and 100 μg/mL streptomycin. Endothelial cell migration assay was set up in transwell 24‐well plates with 8‐μ m pore filters. 1 × 10^5^ cells were seeded per well in the upper chamber after 1‐hour serum starvation. After 12 hours incubation, the cells in the upper surface of each filter were moved using cotton swabs, and the cells migrated into the lower surface were fixed with 4% PFA for 30 minutes and then stained with crystal violet. The cell number was counted in 4 random microscope visual fields in each well.

### 2.6 Tube formation assay

Tube formation assay was performed as reported previously.^29^ Endothelial cell tube formation assay was conducted in 48‐well plates precoated with Matrigel (BD). 1 × 10^5^ cells were seeded per well after 1‐hour serum starvation. After 5‐, 7‐, 9‐ and 12‐hour incubation at 37°C, the tube formation of HMECs was observed and the number of tube branches was quantified by counting 4 random microscope visual fields in each well.

### 2.7 Wound healing assay

Human microvascular endothelial cells or BMSCs were grown to confluency. A linear wound was made by scraping a non‐opening Pasteur pipette across the confluent cell layer. 5, 7, 9 and 12 hours after wound, the migrate cells were observed and measured by counting 4 random microscope visual fields in each well.

### 2.8 qRT‐PCR analysis

Total RNA from cells was extracted using TRIzol reagent (Invitrogen). 1 μg total RNA was used to perform reverse transcription using the PrimeScript RT reagent Kit (Takara). Amplification reactions were set up in 25 μL reaction volumes containing SYBR Green PCR Master Mix (PE Applied Biosystems). Relative quantification was calculated by normalizing the test crossing thresholds (Ct) with the β‐actin amplified control Ct. The results were normalized to β‐actin.

### 2.9 Western blot

Total cell lysates were separated by SDS‐PAGE (sodium dodecyl sulphate polyacrylamide gel electrophoresis) and blotted on polyvinylidene difluoride membranes (Millipore). Then, the membranes were blocked with 5% milk (170‐6404, Bio‐Rad Laboratories, Inc) and incubated with specific antibodies to Klf3 (Invitrogen Antibodies, PA5‐18030, 1:1000), JunB (Cell Signaling Technology, 3753, 1:1000), VEGFA (Proteintech, 19003‐1‐AP, 1:1000) and α‐Tublin (Proteintech, 11224‐1‐AP, 1:2000). Blots were visualized using SuperSignal West Pico PLUS Chemiluminescent Substrate (SD251210, Thermo Fisher Scientific, Inc).

### 2.10 Flow cytometry

We isolated femora and tibia from mice, then crushed the metaphysis region in ice‐cold PBS. Bone pieces were digested using 1 mg/mL type I A collagenase at 37°C for 20 minutes to obtain single‐cell suspensions. Then, the cells were counted and incubated for 45 minutes at 4°C with endomucin antibody (Santa Cruz, SC‐65495, 1:100) and APC‐conjugated CD31 antibody (R&D Systems, FAB3628A, 1:100). We performed acquisition on a fluorescence‐activated cell sorting (FACS) FACScan cytometer (BD Immunocytometry Systems).

### 2.11 μCT analysis

We used the high‐resolution μCT (Skyscan 1172, Bruker microCT, Kontich, Belgium) to perform the µCT analysis. The scanner was set at a voltage of 65 kV, a current of 153 μA and a resolution of 15 μm per pixel. We used the image reconstruction software (NRecon, version 1.6, Bioz, Inc, Palo Alto, CA, USA), data analysis software (CT Analyser, version 1.9, Bruker microCT) and 3‐dimensional model visualization software (μCT Volume, version 2.0, Bruker microCT) to analyse the parameters of the trabecular bone. Trabecular bone volume per tissue volume (Tb. BV/TV) were measured.

### 2.12 Histochemistry

Histochemistry staining was performed as reported previously.^30^, ^31^ Femora were dissected from mice. After fixing overnight with 10% formalin at 4°C, the samples were decalcified at 4°C using 10% EDTA (pH 7.4) for 21 days and then embedded in paraffin. Four‐micrometre‐thick femora were used for staining. The slides were processed for TRAP, and HE staining was performed using a standard protocol (Sigma‐Aldrich).

### 2.13 Immunocytochemistry

Immunocytochemistry staining was performed as reported previously.^32^ Femora were dissected from mice. After fixing overnight with 10% formalin at 4°C, the samples were decalcified at 4°C using 10% EDTA (pH 7.4) for 21 days and then embedded in paraffin. Four‐micrometre‐thick femora were used for staining. The slides were stained with individual primary antibodies to OCN (Takara Bio Inc M137) at 4°C overnight. Horseradish peroxidase‐streptavidin detection system (Dako) was used to detect immunoactivity. Then, we counterstained the sections with haematoxylin (Sigma‐Aldrich).

### 2.14 Immunofluorescence

Immunofluorescence staining was performed as reported previously.^33^, ^34^ Femora were dissected from mice. After fixing with ice‐cold 4% paraformaldehyde solution for 4 hours, the samples were decalcified in 0.5 mol/L EDTA (pH 7.4) at 4℃ for 24 hours (1‐ and 3‐month‐old mice) or for 48 hours (12‐month‐old mice). The bone samples were then incubated in 20% sucrose and 2% polyvinylpyrrolidone (PVP) solution overnight, as described previously.^35^ For CD31^hi^EMCN^hi^ vessels staining, the tissues were embedded in 8% gelatin (porcine) in presence of 20% sucrose and 2% PVP. Forty‐micrometre‐thick bone sections were stained with primary antibodies to mouse CD31 (Abcam, ab28364, 1:100) and endomucin (Santa Cruz, V.7C7, 1:50) overnight at 4°C. For other immunofluorescence staining, we embedded the tissues in optimal cutting temperature compound. Ten‐micrometre‐thick longitudinally oriented bone sections were stained with individual primary antibodies to mouse Alkaline phosphatase (Abcam, ab108337, 1:200) and Osterix (Abcam, ab22552, 1:100,) overnight at 4°C. The slides were stained with secondary antibodies conjugated with fluorescence at room temperature for 1 hour while avoiding light subsequently. We used isotype‐matched controls, such as polyclonal rabbit IgG (R&D Systems, AB‐105‐C), polyclonal goat IgG (R&D Systems, AB‐108‐C) and monoclonal rat IgG2A (R&D Systems, 54447) under the same concentrations and conditions as negative controls.

### 2.15 Statistics

The data are presented as the mean ± SD; For comparisons of two groups, two‐tailed Student's *t* test was used. For comparisons of multiple groups, one‐way ANOVA was used. Differences were considered significant at *P* < .05. No randomization or blinding was used, and no animals were excluded from analysis. Sample sizes were selected on the basis of previous experiments.

### 2.16 Study approval

All animal care protocols and experiments were reviewed and approved by the Animal Care and Use Committees of the Laboratory Animal Research Center at Xiangya Medical School of Central South University.

## 3 RESULTS

### 3.1 The amount of CD31^hi^EMCN^hi^ vessels decreased during ageing but increased in bone regeneration

**Figure 1:**
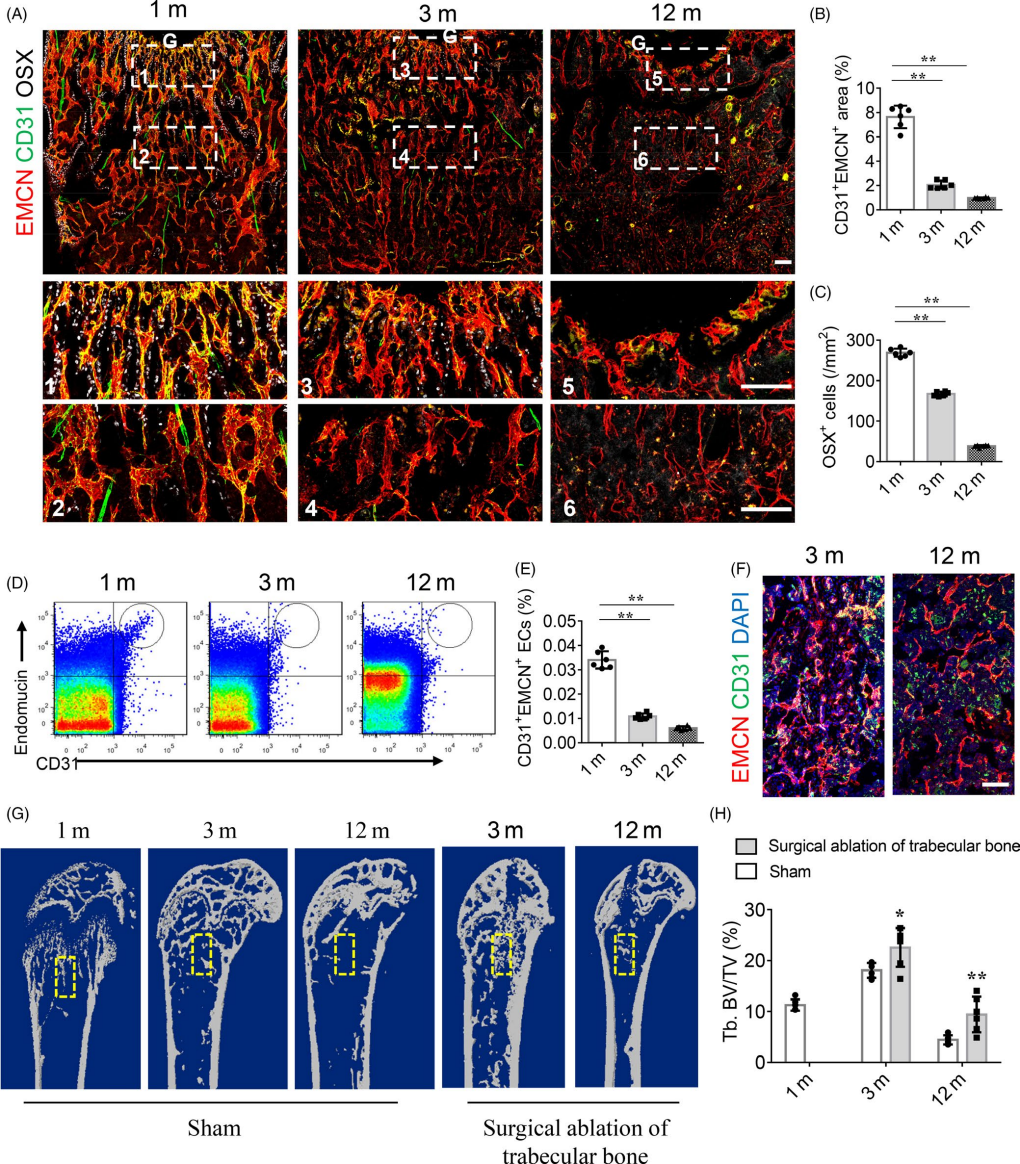
CD31^hi^Emcn^hi^ vessel and bone formation decreased during ageing but increased in bone regeneration. A, Representative images of CD31 (green), EMCN (red) and Osterix (white) immunostaining. Scale bar, 100 μm. G, growth plate. B, Quantification of CD31 and EMCN positive vessel volume in distal femora. C, Quantitative analysis of Osterix‐positive (OSX^+^) osteoprogenitors in distal femora. D and E, FACS analysis dot plot (D) and quantification (E) of CD31^hi^EMCN^hi^ ECs. F, Representative images of CD31 (green) and EMCN (red) immunostaining in bone regeneration area after femoral trabecular bone ablation. Nuclei, DAPI (blue). Scale bar, 100 μm. G and H, Representative μCT images (G) and quantitative μCT analysis (H) of bone regeneration after femoral trabecular bone ablation. Selected areas for the measurements of bone volume (BV)/tissue volume (TV) were indicated with a yellow square. Data are shown as mean ± SD, (n = 6 in B, C, E and H). *P < .05; **P < .01 by one‐way ANOVA

As animals age, the decrease in the number of osterix‐positive (OSX^+^) osteoprogenitors and bone mass correlates with the pronounced reduction in CD31^hi^Emcn^hi^ vessels (Figure 1A‐C and G‐H). The amount of CD31^hi^Emcn^hi^ vessels, which mainly appear in the metaphysis just below the growth plate in 1 month old, was significantly decreased in adults (3 months old) and was nearly absent in aged (12 months old) mice (Figure 1A,B). Flow cytometry analysis confirmed the age‐dependent reduction of CD31^hi^Emcn^hi^ endothelial cells (Figure 1D,E). To investigate the role of CD31^hi^EMCN^hi^ vessels in bone regeneration, we constructed a bone regeneration model after surgical ablation of the trabecular bone.^24^, ^25^ We found that abundant CD31^hi^EMCN^hi^ vessels, as well as alkaline phosphatase‐positive (ALP^+^) osteoprogenitors and OSX^+^ osteoprogenitors, emerged in the bone regeneration area in the 3‐month‐old mice at 1 week after surgery (Figure 1F and Figure S1). The amount of CD31^hi^EMCN^hi^ vessels in the femur of the 12‐month‐old mice was also increased at 1 week after trabecular bone ablation (Figure 1F). The bone volume in the regeneration area was increased together with the number of CD31^hi^EMCN^hi^ vessels and osteoprogenitors cells (Figure 1G‐H and Figure S1). These results indicated that the CD31^hi^EMCN^hi^ vessels might play an important role in bone regeneration.

### 3.2 Endothelial‐specific Klf3 knockout mice show increased numbers CD31^hi^EMCN^hi^ vessel and accelerated bone regeneration

**Figure 2:**
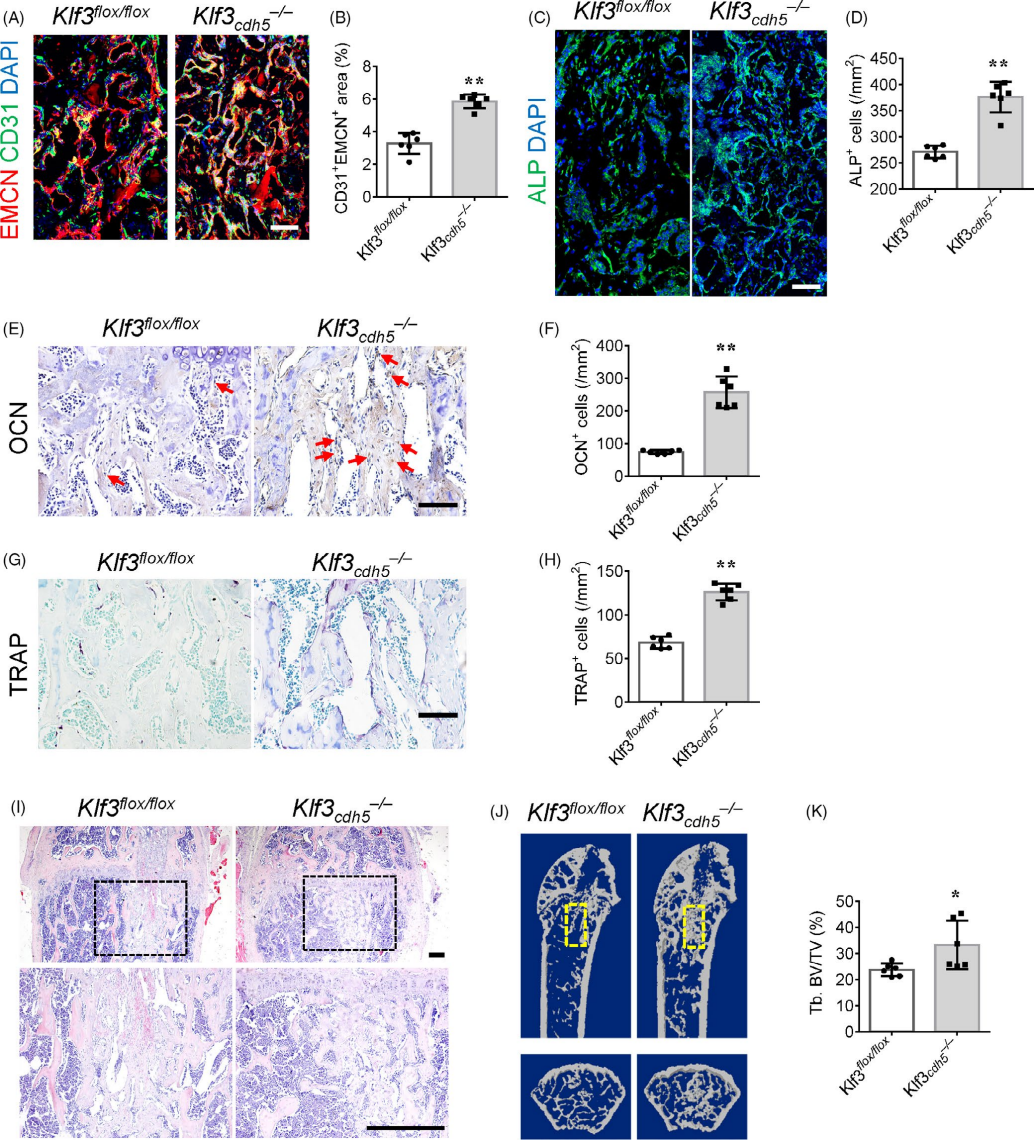
Endothelial‐specific *Klf3* knockout mice show increased CD31^hi^EMCN^hi^ vessels and accelerated bone regeneration. A and B, Representative images (A) and quantification (B) of CD31 (green) and EMCN (red) immunostaining in bone regeneration area after femoral trabecular bone ablation of 3‐month‐old mice. Nuclei, DAPI (blue). Scale bar, 100 μm. C and D, Representative images (C) and quantification (D) of alkaline phosphatase (ALP, green) immunostaining in bone regeneration area of 3‐month‐old mice. Nuclei, DAPI (blue). Scale bar, 100 μm. E and F, Immunohistochemical staining (E) and quantification (F) of osteocalcin positive cells (OCN^+^, brown) in bone regeneration area of 3‐month‐old mice. Red arrows point at positive cells. Scale bar, 100 μm. G and H, Representative images (G) and quantification (H) of TRAP (red) staining in bone regeneration area of 3‐month‐old mice. Scale bar, 100 μm. I, Representative images of HE staining in distal femora of 3‐month‐old mice. Scale bar, 200 μm. J and K, Representative μCT images (J) and quantitative μCT analysis (K) of bone regeneration after femoral trabecular bone ablation of 3‐month‐old mice. Selected areas for the measurements of bone volume (BV)/tissue volume (TV) were indicated with a yellow square. Data are shown as mean ± SD. (n = 6 in B, D, F, H and K). *P < .05; **P < .01 by two‐tailed Student's *t* test

Krüppel like factor 3 is a potent transcriptional repressor with diverse roles in differentiation. Our previous research demonstrated that specific knockout of *Klf3* in endothelial cells could significantly promote CD31^hi^EMCN^hi^ vessel formation in bone tissues.^23^ To investigate the role of CD31^hi^EMCN^hi^ vessels in bone regeneration in vivo, we crossed Cdh5 (PAC)‐Cre transgenic mice with *Klf3^flox/flox^* mice to specifically knock out *Klf3* in endothelial cells (*Klf3_cdh5_^−/−^*). The *Klf3_cdh5_^−/−^* mice and their *Klf3^flox/flox^* littermates were subjected to surgical ablation of the trabecular bone to generate the bone regeneration model at 3 months old and 12 months old. One week after surgery, femurs of these mice were collected to detect CD31^hi^EMCN^hi^ vessels and bone formation. Co‐immunostaining of CD31 and endomucin (EMCN) identified higher levels of the CD31^hi^EMCN^hi^ endothelium and increased numbers of ALP^+^ and OSX^+^ osteoprogenitors in the bone regeneration area of 3‐month‐old *Klf3_cdh5_^−/−^* mice compared with that of their age‐matched *Klf3^flox/flox^* littermates (Figure 2A‐D and Figure S2A‐B). The numbers of osteoblasts and osteoclasts were increased in the bone regeneration area of the *Klf3_cdh5_^−/−^* mice (Figure 2E‐H), which indicated an increase in bone modelling and remodelling. Microcomputed tomography (μCT) and haematoxylin and eosin (HE) staining showed significantly increased bone regeneration in Klf3_cdh5_^−/−^ mice compare with that in their *Klf3^flox/flox^* littermates (Figure 2I‐K). We also found increased CD31^hi^EMCN^hi^ endothelium formation and bone regeneration in 12‐month‐old *Klf3_cdh5_^−/−^* mice compared with those in their age‐matched *Klf3^flox/flox^* littermates at 1 week after surgical ablation of the trabecular bone (Figure S2C‐F). These results indicated that promoting the formation of CD31^hi^EMCN^hi^ vessels by specific knockout *Klf3* in endothelia cells could stimulate bone regeneration.

### 3.3 Ophiopogonin D acts as a KLF3 inhibitor and promotes vessels formation in vitro

**Figure 3:**
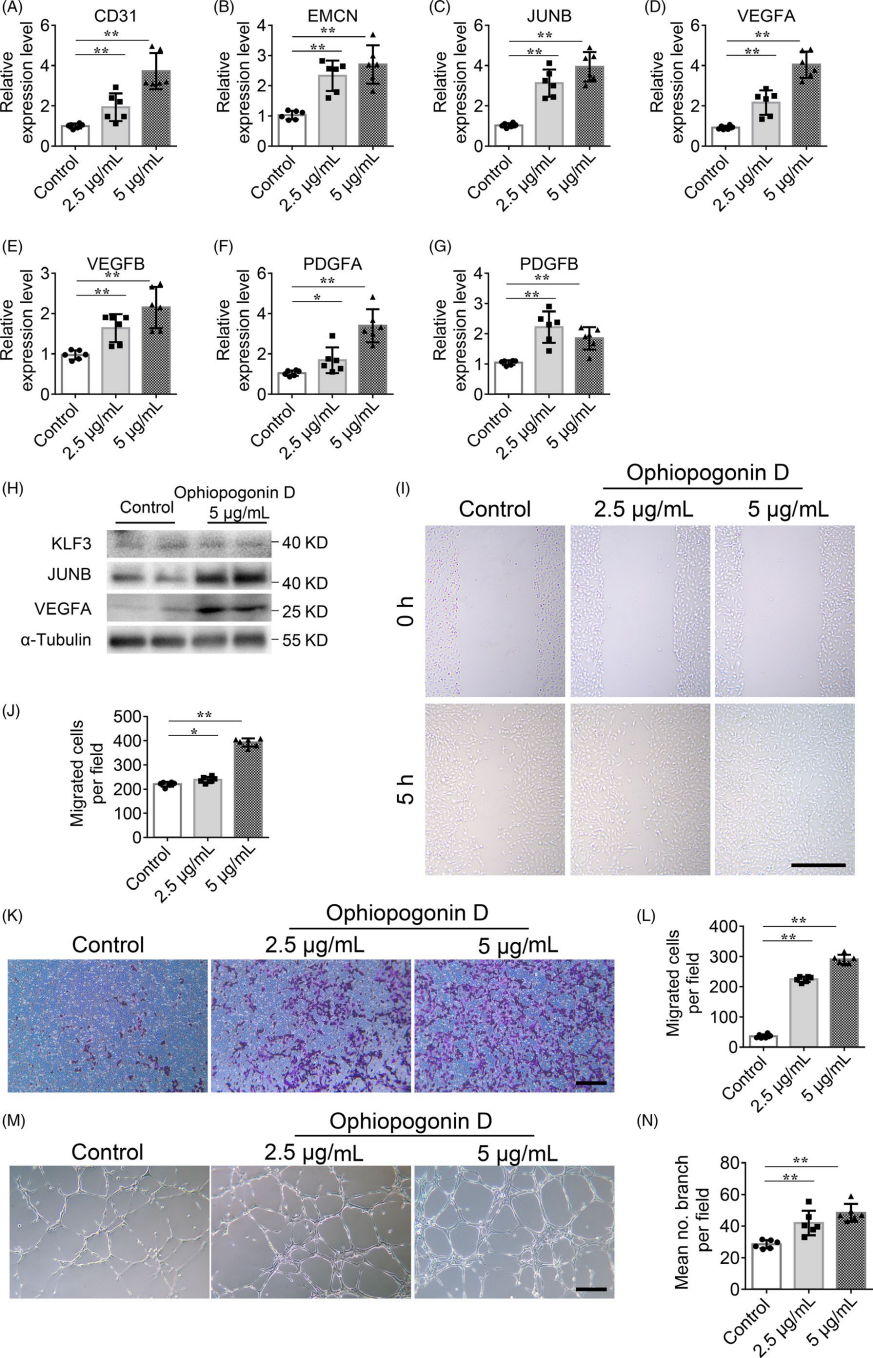
Ophiopogonin D acts as a KLF3 inhibitor and promotes vessel formation in vitro. A‐G, qRT‐PCR analysis of the relative levels of *CD31* (A), *EMCN* (B), *JUNB* (C), *VEGFA* (D), *VEGFB* (E), *PDGFA* (F) and *PDGFB* (G). H, Western blotting analysis of the KLF3, JUNB and VEGFA expression in HMECs treated with vehicle or different doses of ophiopogonin D. I and J, Representative images (I) and quantification (J) of migration HMECs in wound healing assay. Scale bar, 500 μm. K‐L, Representative images (K) and quantification (L) of a transwell migration assay. Scale bar, 200 μm. M and N, Representative images (M) and quantification (N) of tube branch numbers of a matrigel tube formation assay. Scale bar, 200 μm. Data are shown as mean ± SD, (The cell experiments were repeated for three times, n = 6 in A‐G, J, L and N). *P < .05; **P < .01 by one‐way ANOVA

Our previous research demonstrated that KLF3 represses the expression of *JUNB* (JunB proto‐oncogene, AP‐1 transcription factor subunit) as well as its downstream factor *VEGFA* (vascular endothelial growth factor A) in endothelial cells, and further inhibited the process of angiogenesis.^23^ We also identified a natural compound from Radix *Ophiopogon japonicus*, ophiopogonin D, which acts as a KLF3 inhibitor to abolish the transcriptional repression function of KLF3 and further increase the expression of *JUNB* and *VEGFA* in endothelial cells.^23^ As expected, we observed that *CD31*, *EMCN*, and *JUNB* and vessel growth factors (*VEGFA*, *VEGFB*, *PDGFA* (platelet derived growth factor subunit A), and *PDGFB*) transcripts were expressed at higher level in human microvascular endothelial cells (HMECs) treated with ophiopogonin D compared with that in DMSO control treated HMECs (Figure 3A‐G). Chromatin immunoprecipitation‐PCR (ChIP‐PCR) assays showed that ophiopogonin D treatment affected the binding of KLF3 to the promoter of *JUNB*.^23^ Western blotting also showed increased protein levels of JUNB and VEGFA in HMECs treated with ophiopogonin D, without affect the expression of KLF3 (Figure 3H). We further confirmed the angiogenesis stimulating function of ophiopogonin D using migration and tube formation assays. The results showed that ophiopogonin D treatment could increase the migration and tube formation ability of HMECs (Figure 3I‐N). These data demonstrated that ophiopogonin D works as a KLF3 inhibitor and has a positive effect on angiogenesis in vitro.

### 3.4 Ophiopogonin D does not affect BMSC migration and osteoblastic differentiation in vitro

**Figure 4:**
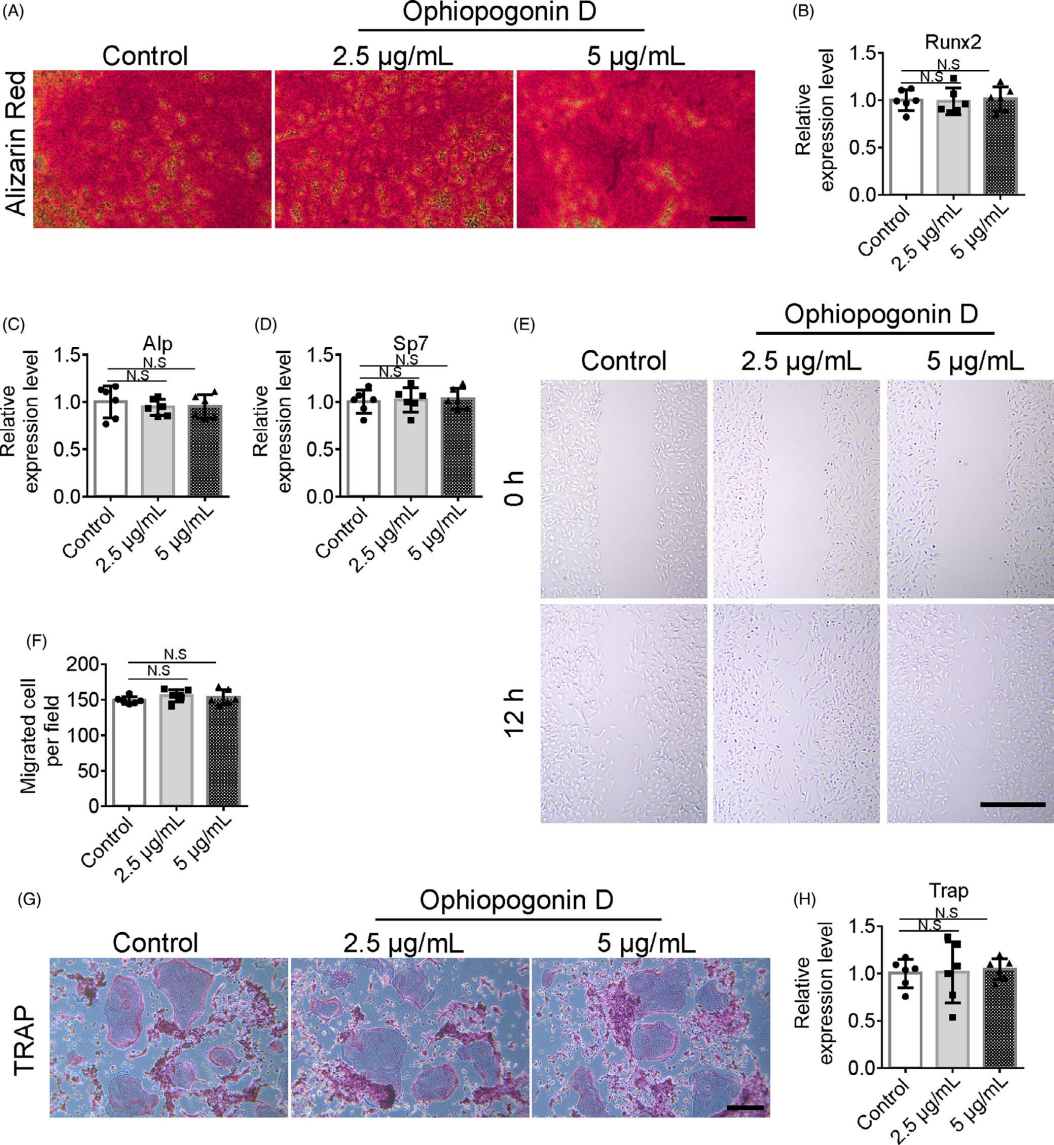
Ophiopogonin D do not affect BMSC migration and osteoblastic differentiation in vitro. A, Representative images of Alizarin Red S staining in BMSCs treated with or without ophiopogonin D under osteogenic induction. Scale bar, 100 μm. B‐D, qRT‐PCR analysis of the relative levels of *Runx2* (B), *Alp* (C) and *Sp7* (D). E and F, Representative images (E) and quantification (F) of migration BMSCs in wound healing assay. Scale bar, 500 μm. G, Images of TRAP staining of bone marrow monocytes treated with 30 ng mL^−1^ of M‐CSF, 200 ng mL^−1^ of RANKL and with or without ophiopogonin D for 8 d. Scale bar, 200 μm. H, qRT‐PCR analysis of the relative levels of *Trap*. (The cell experiments were repeated for three times, n = 6 in B‐D and F‐H). N.S, no significance by one‐way ANOVA

To investigate the role of ophiopogonin D in bone formation in vitro, we isolated primary bone marrow‐derived stem cells (BMSCs) and carried out migration and osteogenic differentiation assays. Alizarin Red staining showed no difference between the ophiopogonin D and DMSO control treatment groups in the BMSC osteogenic differentiation assays (Figure 4A). The expression levels of the osteoblastic markers *Runx2* (RUNX family transcription factor 2), *Alp* (alkaline phosphatase) and *Sp7* (SP7 transcription factor) remained unchanged after ophiopogonin D treatment (Figure 4B‐D). The results of BMSC migration assays indicated that ophiopogonin D could not promote BMSCs migration (Figure 4E, F). Ophiopogonin D also did not affect osteoclast differentiation, as indicated by the evaluation of tartrate‐resistant acid phosphatase (TRAP) staining and the expression levels of the osteoclast transcription factors *Trap* of bone marrow monocytes and macrophages, which underwent stimulation of osteoclast differentiation (Figure 4G, H). This result indicated that Ophiopogonin D treatment does not affect BMSC migration, osteoblastic differentiation and osteoclast differentiation in vitro.

### 3.5 Ophiopogonin D treatment promotes CD31^hi^EMCN^hi^ vessel and bone formation during bone regeneration

**Figure 5:**
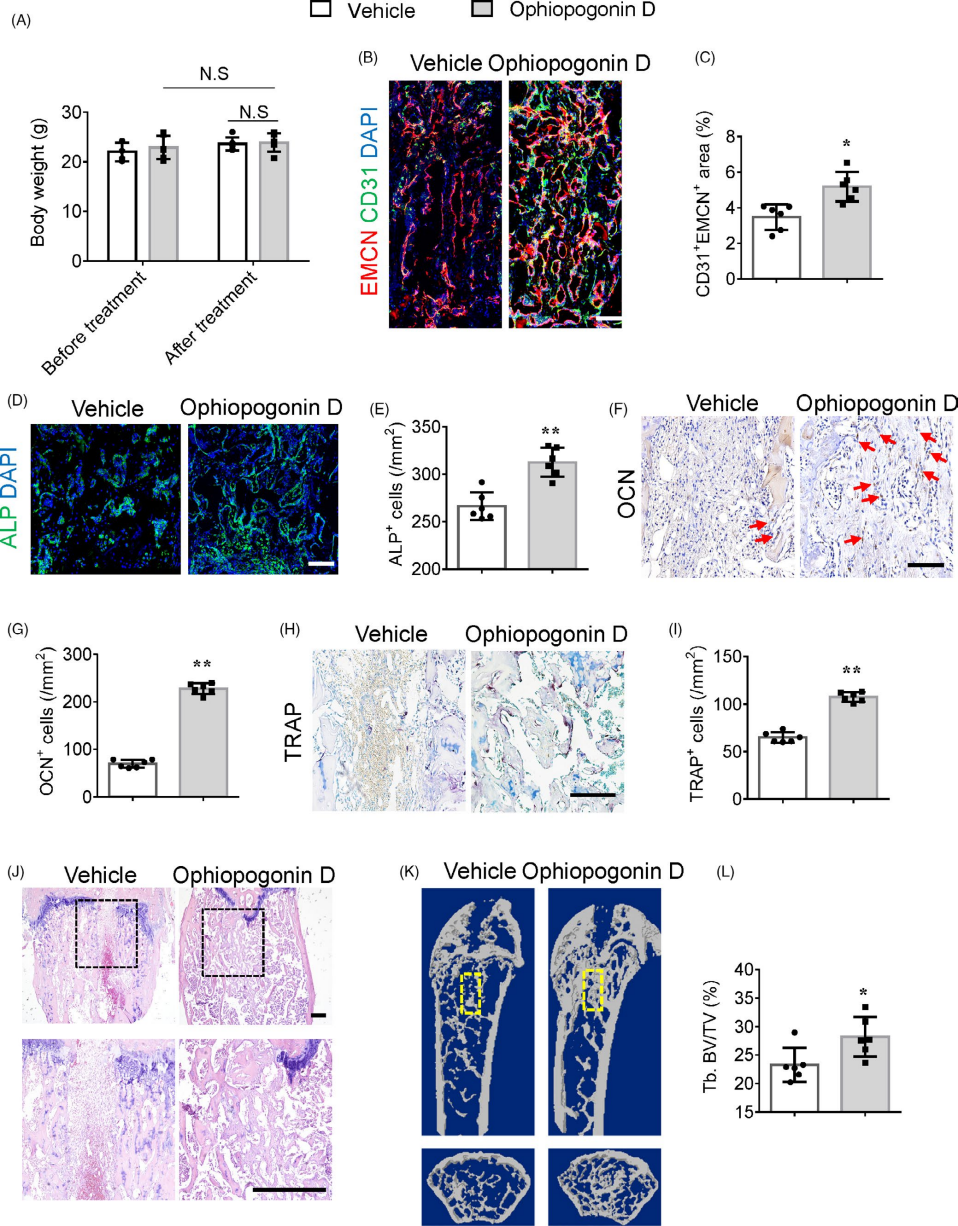
Ophiopogonin D treatment promotes CD31^hi^EMCN^hi^ vessel formation and bone regeneration. A, The body weight of 3‐month‐old mice after surgical ablation of trabecular bone and further treated with ophiopogonin D and vehicle controls. B and C, Representative images (B) and quantification (C) of CD31 (green) and EMCN (red) immunostaining in bone regeneration area of 3‐month‐old mice after femoral trabecular bone ablation. Nuclei, DAPI (blue). Scale bar, 100 μm. D and E, Representative images (D) and quantification (E) of alkaline phosphatase (ALP, green) immunostaining in bone regeneration area of 3‐month‐old mice. Nuclei, DAPI (blue). Scale bar, 100 μm. F and G, Immunohistochemical staining (F) and quantification (G) of osteocalcin positive cells (OCN^+^, brown) in bone regeneration area of 3‐month‐old mice. Red arrows point at positive cells. Scale bar, 100 μm. H and I, Representative images (H) and quantification (I) of TRAP (red) staining in bone regeneration area of 3‐month‐old mice. Scale bar, 100 μm. J, Representative images of HE staining in distal femora of 3‐month‐old mice. Scale bar, 200 μm. K and L, Representative μCT images (K) and quantitative μCT analysis (L) of bone regeneration after femoral trabecular bone ablation of 3‐month‐old mice. Selected areas for the measurements of bone volume (BV)/tissue volume (TV) were indicated with a yellow square. Data are shown as mean ± SD. (n = 6 in A, C, E, G, I and L). *P < .05; **P < .01; N.S, no significance by one‐way ANOVA or two‐tailed Student's *t* test

To investigate whether treatment with ophiopogonin D could promote CD31^hi^EMCN^hi^ vessel formation and further stimulate bone regeneration in vivo, 3‐month‐old and 12‐month‐old C57BL/6J mice, after surgical ablation of the trabecular bone, were treated intraperitoneally with ophiopogonin D at 20 mg/kg every day for 1 week. This treatment did not affect the body weight of either group of mice (Figure 5A and Figure S3A). Treatment with ophiopogonin D increased the amount of CD31^hi^EMCN^hi^ endothelium in the regeneration area of both 3‐month‐old and 12‐month‐old mice compared with that in vehicle‐treated mice, as detected by immunofluorescence staining (Figure 5B,C and Figure S3B,C). The number of ALP^+^ osteoprogenitors, OSX^+^ osteoprogenitors, OCN^+^ osteoblasts and TRAP^+^ osteoclasts in the regeneration area also increased after ophiopogonin D treatment in 3‐month‐old mice, which indicated increased bone modelling (Figure 5D‐I and Figure S3D,E). HE staining and μCT confirmed the significantly increased bone regeneration in the ophiopogonin D‐treated mice compared with that in the vehicle‐treated control mice in both the 3‐month‐old and 12‐month‐old groups (Figure 5J‐L and Figure S3F,G). Taken together, these results suggested that ophiopogonin D treatment could promote CD31^hi^EMCN^hi^ vessel formation and further accelerate bone regeneration.

## 4 DISCUSSION

Blood vessels play an important role in tissue regeneration by recruiting progenitor cells.^36^, ^37^, ^38^, ^39^ In skeleton tissue, CD31^hi^EMCN^hi^ vessels generate distinct metabolic and molecular microenvironments, maintain perivascular osteoprogenitors and couple angiogenesis to osteogenesis.^18^, ^40^ Osteoblast derived Slit guidance ligand 3 (SLIT3) could increase the formation of the CD31^hi^EMCN^hi^ endothelium. Pre‐osteoclasts could induce the CD31^hi^EMCN^hi^ vessel subtype, subsequently preventing bone loss in osteoporosis.^21^ Our previous study revealed that the microRNA miR‐497∼195 cluster contributes to the increase in CD31^hi^EMCN^hi^ vessels and bone formation in aged mice.^22^ However, few studies have investigated the role of CD31^hi^EMCN^hi^ vessels in bone regeneration. The results of the present study showed that many CD31^hi^EMCN^hi^ vessels emerged after bone injury in mice at the age at which CD31^hi^EMCN^hi^ vessels are nearly absent physiologically. Furthermore, promoting CD31^hi^EMCN^hi^ vessel formation could stimulate bone regeneration in mice. These results indicated the important role of CD31^hi^EMCN^hi^ vessels, not only in bone formation, but also in bone regeneration.

Krüppel like factor 3, which is a potent transcriptional repressor that mediates transcriptional silencing via recruiting the corepressor C‐terminal binding protein (CTBP), could modulate diverse physiological processes in various tissues, such as angiogenesis, B lymphopoiesis, adipogenesis, erythropoiesis, myogenesis and cardiac development.^41^, ^42^, ^43^, ^44^, ^45^ *JunB* is one of the KLF3 downstream genes and acts as a critical regulator of *Vegfa*.^46^, ^47^, ^48^ Our previous studies have shown that KLF3 inhibited the expression of *JUNB* and *VEGFA,* and further repressed angiogenesis.^23^ We also demonstrated that specific knockout of *Klf3* in endothelial cells increased the formation of CD31^hi^EMCN^hi^ vessels and increased bone formation.^23^ Here, we extended our study and confirmed that specific knockout of *Klf3* in endothelial cells also could stimulate CD31^hi^EMCN^hi^ vessel formation after bone injury and could accelerate bone regeneration.

Ophiopogonin D is a natural compound isolated from the traditional Chinese herbal agent Radix *Ophiopogon japonicus*,^49^ which showed anti‐osteoporotic effects in a murine ovariectomized (OVX) model.^50^ Previously, we identified that ophiopogonin D could bind to KLF3 and suppress its function, thus further promoting angiogenesis.^23^ In the present study, we further demonstrated that ophiopogonin D also could increase CD31^hi^EMCN^hi^ vessel formation and bone regeneration after bone injury.

In summary, we revealed the important role of CD31^hi^EMCN^hi^ vessels in bone regeneration and demonstrated the natural compound ophiopogonin D could increase the number of CD31^hi^EMCN^hi^ vessels and promoted bone regeneration after bone injury. Our findings provide a potentially novel strategy to accelerate bone healing.

## CONFLICT OF INTEREST

The authors have declared that no conflict of interest exists.

## AUTHOR CONTRIBUTIONS

TJ.J and M.Y designed the experiments; M.Y carried out most of the experiments, generated data and drafted the manuscript; Q.G, Y.X, Y.H, CJ.L and T.S helped to collect the samples. TJ.J, XH.L and M.Y proofread the manuscript; TJ.J supervised the experiments, analysed results, co‐wrote the manuscript, and is the guarantor of this work and has full access to all the data in this study and takes the responsibility for data accuracy.

## Data Availability

The data that support the findings of this study are available within the article and Supplementary Files or available from the authors upon request.
