# Supplementary figures and images for: Ophiopogonin D promotes bone regeneration by stimulating CD31hiEMCNhi vessel formation
Source: Cell Prolif. 2020 Feb 20;53(3):e12784. doi: 10.1111/cpr.12784 (PMC7106967; doi:10.1111/cpr.12784)

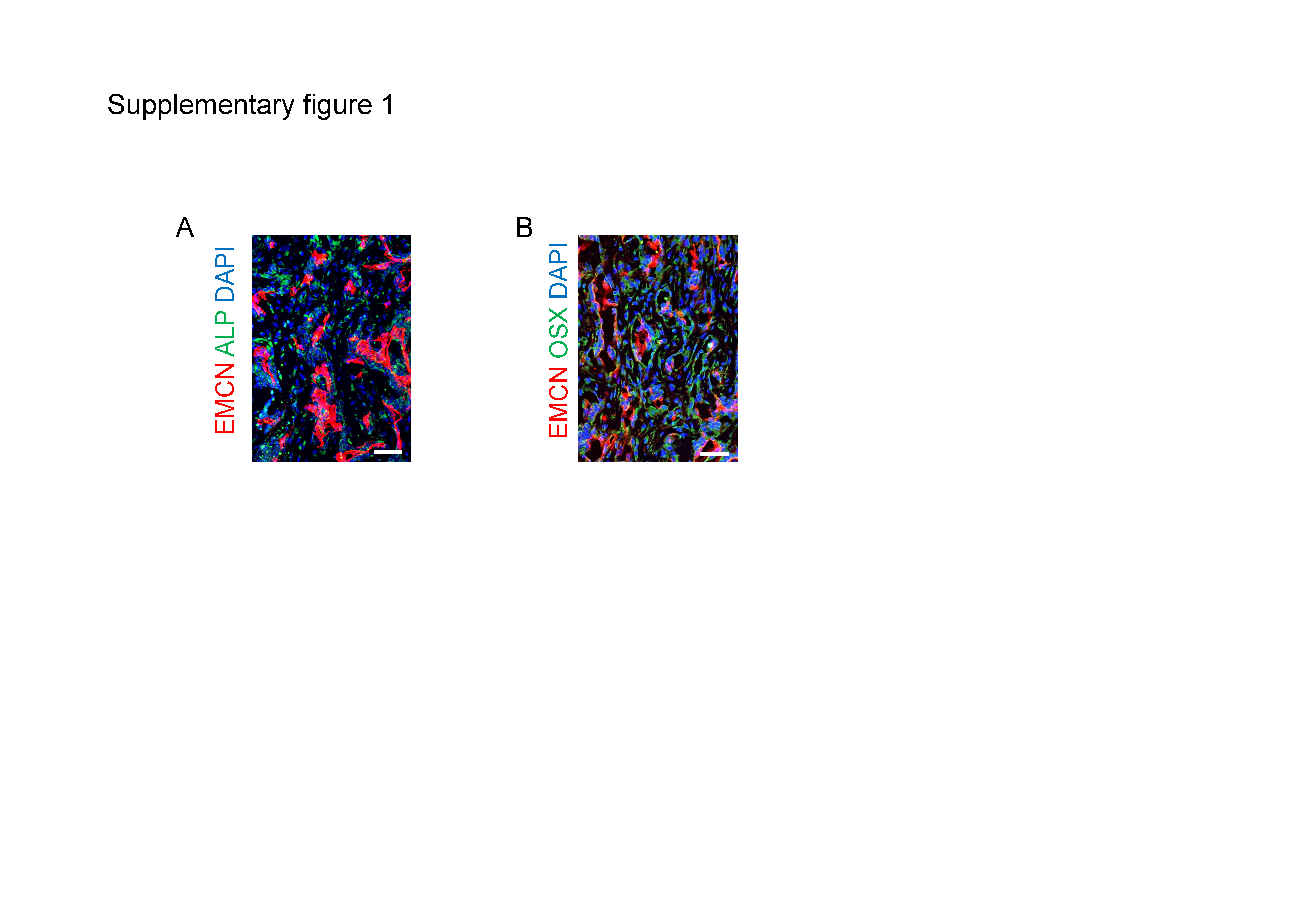

Supplement: Supplementary file 1 [file CPR-53-e12784-s001.tif]

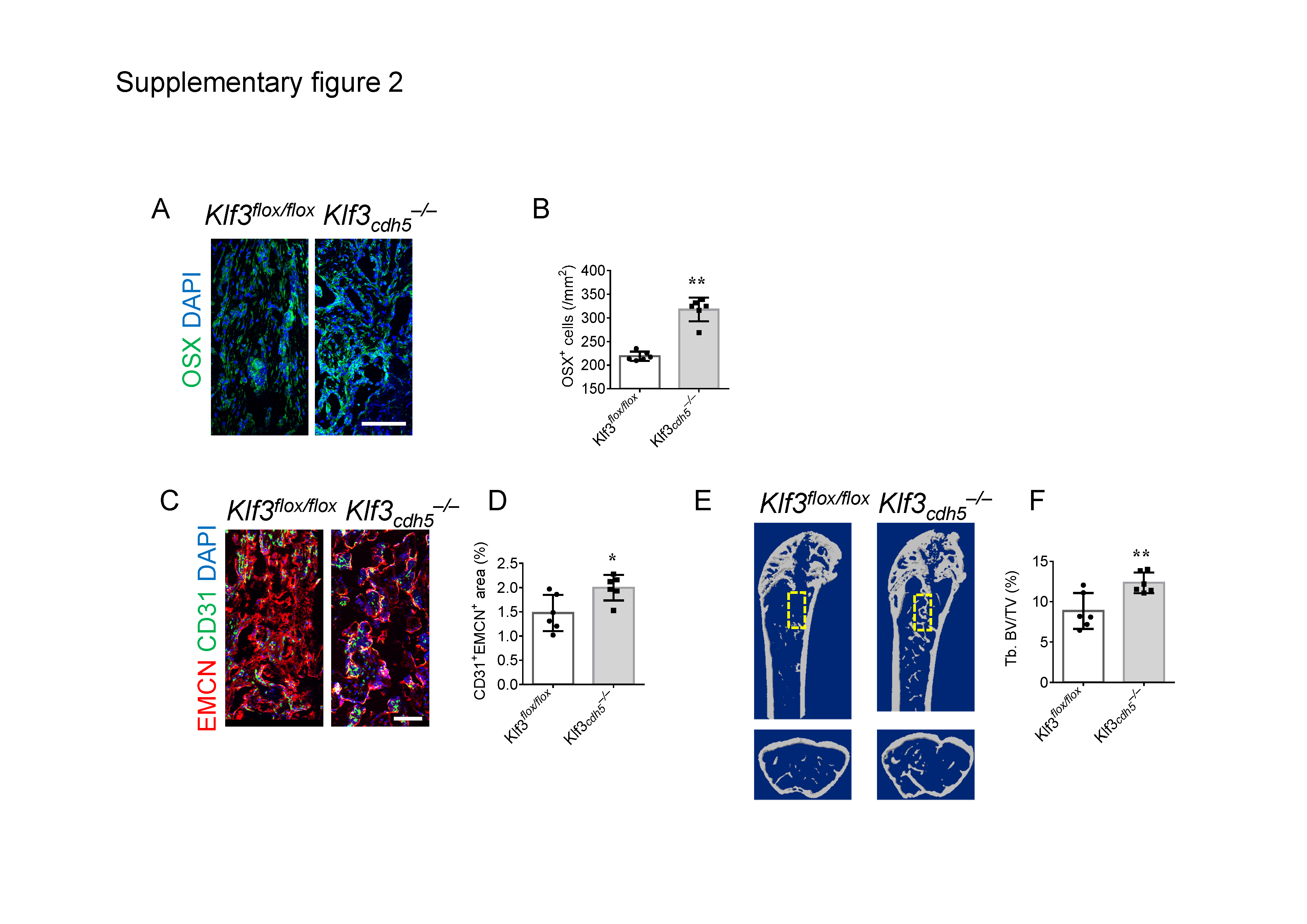

Supplement: Supplementary file 2 [file CPR-53-e12784-s002.tif]

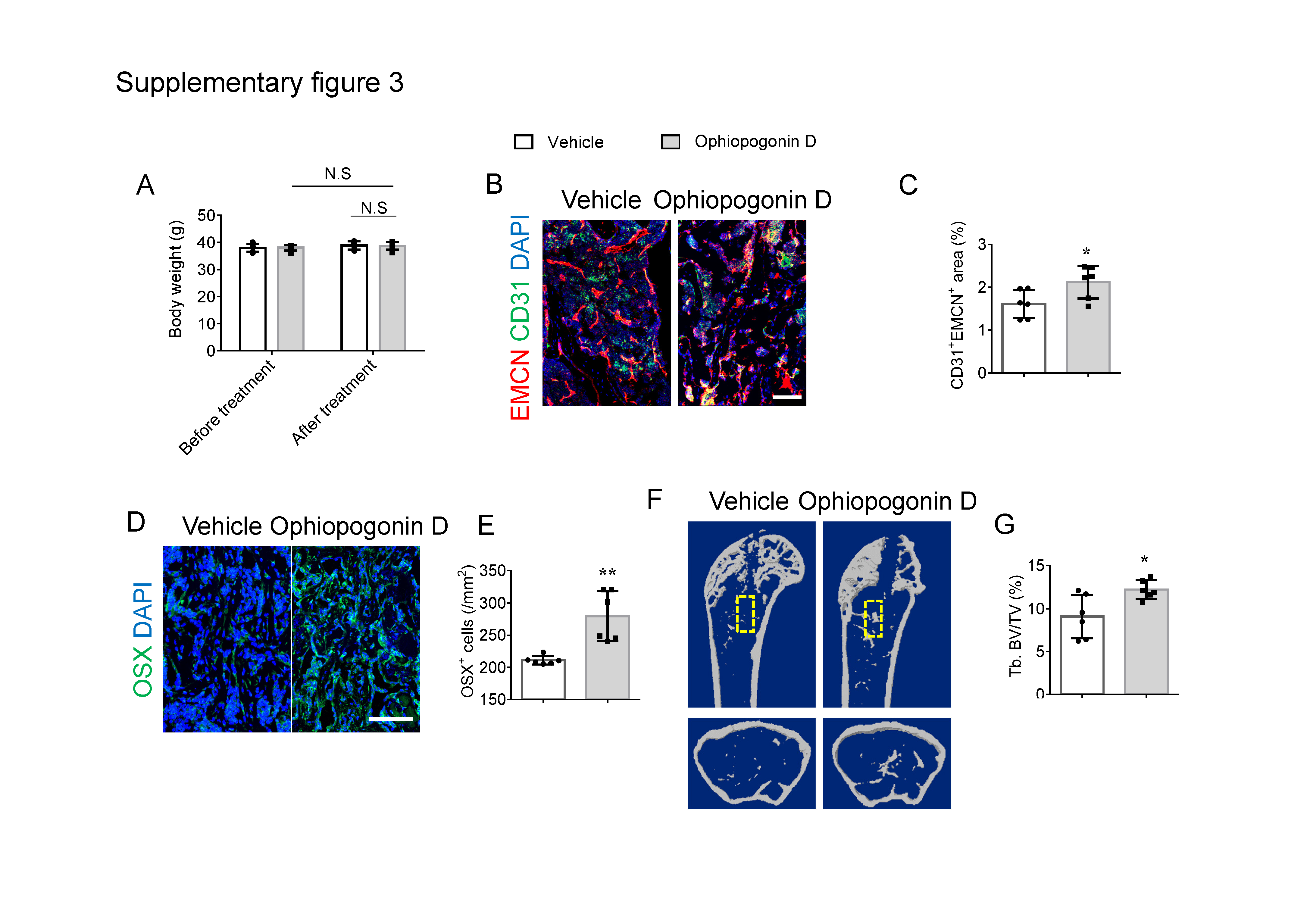

Supplement: Supplementary file 3 [file CPR-53-e12784-s003.tif]
