# Supplementary material for: Ophiopogonin D promotes bone regeneration by stimulating CD31hiEMCNhi vessel formation
Source: Cell Prolif. 2020 Feb 20;53(3):e12784. doi: 10.1111/cpr.12784 (PMC7106967; doi:10.1111/cpr.12784)
Supplement: Supplementary file 4 [file CPR-53-e12784-s004.docx]

**Supplementary figure legends**

**Supplementary figure 1. Abundant osteoprogenitors emerged with CD31^hi^EMCN^hi^ vessels in bone regeneration area.**

(A) Representative images of EMCN (red) and alkaline phosphatase (ALP, green) immunostaining in bone regeneration area. Nuclei, DAPI (blue). Scale bar, 50 μm. (B) Representative images of EMCN (red) and Osterix (OSX, green) immunostaining in bone regeneration area. Nuclei, DAPI (blue). Scale bar, 50 μm.

**Supplementary figure 2. Endothelial-specific *Klf3* knockout mice show increased CD31^hi^EMCN^hi^ vessels and accelerated bone regeneration at 12-month-old.**

(A-B) Representative images (A) and quantification (B) of osterix (OSX, green) immunostaining in bone regeneration area of 3-month-old mice. Nuclei, DAPI (blue). Scale bar, 100 μm. (C-D) Representative images (C) and quantification (D) of CD31 (green) and EMCN (red) immunostaining in bone regeneration area of 12-month-old mice after femoral trabecular bone ablation. Nuclei, DAPI (blue). Scale bar, 100 μm. (E-F) Representative μCT images (E) and quantitative μCT analysis (F) of bone regeneration after femoral trabecular bone ablation of 12-month-old mice. Selected areas for the measurements of bone volume (BV)/tissue volume (TV) were indicated with a yellow square. Data are shown as mean ± SD. (*n*=6 in B, D and F). **P* < 0.05; ***P* < 0.01 by two-tailed Student’s t test.

**Supplementary figure 3. Ophiopogonin D treatment promotes CD31^hi^EMCN^hi^ vessel and bone formation in bone regeneration are in 12-month-old mice.**

(A) The body weight of 12-month-old mice after surgical ablation of trabecular bone and further treated with ophiopogonin D and vehicle controls for 1 week. (B-C) Representative images (B) and quantification (C) of CD31 (green) and EMCN (red) immunostaining in bone regeneration area of 12-month-old mice after femoral trabecular bone ablation. Nuclei, DAPI (blue). Scale bar, 100 μm. (D-E) Representative images (D) and quantification (E) of osterix (OSX, green) immunostaining in bone regeneration area of 3-month-old mice. Nuclei, DAPI (blue). Scale bar, 100 μm. (F-G) Representative μCT images (F) and quantitative μCT analysis (G) of bone regeneration after femoral trabecular bone ablation of 12-month-old mice with ophiopogonin D or vehicle treatment. Selected areas for the measurements of bone volume (BV)/tissue volume (TV) were indicated with a yellow square. Data are shown as mean ± SD. (*n*=6 in A, C, E and G). **P* < 0.05; ***P* < 0.01; N.S, no significance by one-way ANOVA or two-tailed Student’s t test.
